# Geometric morphometrics for the study of facial expressions in non-human animals, using the domestic cat as an exemplar

**DOI:** 10.1038/s41598-019-46330-5

**Published:** 2019-07-08

**Authors:** Lauren R. Finka, Stelio P. Luna, Juliana T. Brondani, Yorgos Tzimiropoulos, John McDonagh, Mark J. Farnworth, Marcello Ruta, Daniel S. Mills

**Affiliations:** 1 School of Life Sciences, Joseph Bank Laboratories, University of Lincoln, Lincoln, LN6 7DL UK; 2 Animal, Rural and Environmental Sciences, Nottingham Trent University, Southwell, NG25 0QF UK; 3 School of Veterinary Medicine and Animal Science, São Paulo State University (Unesp), São Paulo, 18618-970 Brazil; 4 School of Computer Science, University of Nottingham, Nottingham, NG8 1BB UK

**Keywords:** Animal behaviour, Animal physiology

## Abstract

Facial expression is a common channel for the communication of emotion. However, in the case of non-human animals, the analytical methods used to quantify facial expressions can be subjective, relying heavily on extrapolation from human-based systems. Here, we demonstrate how geometric morphometrics can be applied in order to overcome these problems. We used this approach to identify and quantify changes in facial shape associated with pain in a non-human animal species. Our method accommodates individual variability, species-specific facial anatomy, and postural effects. Facial images were captured at four different time points during ovariohysterectomy of domestic short haired cats (n = 29), with time points corresponding to varying intensities of pain. Images were annotated using landmarks specifically chosen for their relationship with underlying musculature, and relevance to cat-specific facial action units. Landmark data were subjected to normalisation before Principal Components (PCs) were extracted to identify key sources of facial shape variation, relative to pain intensity. A significant relationship between PC scores and a well-validated composite measure of post-operative pain in cats (UNESP-Botucatu MCPS tool) was evident, demonstrating good convergent validity between our geometric face model, and other metrics of pain detection. This study lays the foundation for the automatic, objective detection of emotional expressions in a range of non-human animal species.

## Introduction

Neonates, unlike verbally-capable humans, cannot self-report distressing experiences. Recognising these experiences through other communication channels, such as facial expression, is therefore particularly important^1^. This has fuelled interest in the use of computer-aided facial analysis as a means of automating pain detection^2^, and more widely in the diagnosis of human medical conditions^3^. Non-human animals are similarly limited in their ability to communicate suffering. Since initial reports of distinct pain-linked expressions in mice^4^, there has been growing interest in the development of tools to detect facial expressions of pain in a range of species.

This has led to the creation and varying degrees of validation (and in one instance automation)^5^ of facial pain detection tools in rats^6^, rabbits^7^, horses^8,9^, cats^10,11^, pigs^12^, sheep^5,13^, and ferrets^14^. In their development, protocols have usually involved comparing the facial expressions of subjects (from static images) before and after experimentally induced pain, or those with and without a pre-existing painful condition. Differences in facial expression are then classified in terms of ‘action units’, with various photographic^4,8,13,14^ and illustrative scales^9–11^ generated to depict expressions associated with pain, as well as its supposed intensity.

However, a major limitation of these studies is their dependence on relatively subjective, human-based visual discriminations for quantification. With the exception of one recent study in ferrets^14^, the action units reported have relied upon extrapolation from human systems (i.e. the human Facial Action Coding System (FACS))^15^. In so doing, these studies appropriate the descriptors developed to quantify changes in human facial muscles, and therefore lack an understanding of species-specific underlying facial musculature, and the associated repertoire of movements. This is potentially problematic given that the facial expression of similar emotional states, and associated musculature, can differ between humans and non-human animals^16^. For example, both domestic dogs and humans display consistent, distinctive facial expressions in response to emotive stimuli^16^. However, in emotionally comparable contexts, different kinds of facial expressions are produced by each species^16^. This is further confounded by interspecies variability in the specific muscles involved in the production of visually similar action units, as well as by differences in the actual repertoire of facial action units available (e.g. see^15,17–19^).

Recent progress has been made regarding both the automated localisation of facial landmarks, and the detection of facial action units anticipated to be associated with pain in sheep^5^. However, this approach used a ‘histogram of orientated gradients’ method, where a priori assumptions about the specific action units of interest were made. These action units were not species specific, nor were they defined in relation to underling musculature (see^5^ and^13^). Accordingly, these previous reports are subject to a range of potential anthropocentric biases, including the expressions attended to, and their subsequent interpretation.

The facial action units currently in use in the literature may represent the most commonly observed variations in facial expressions (e.g.^15,17–23^). However, due to the inherent complexity of facial musculature, they are unlikely to be exhaustive. As a result, they may not always capture all facial expressions of relevance to a specific research question. For example, some facial expressions may only occur in a specific species, and only in a specific context. Additionally, action units should be coded dynamically, with units representing the process of movement or muscle constriction, rather than the more global impact of their outputs. Thus, action units are not designed to capture facial expressions which have a more static quality, such as those that might arise from an ongoing state. Accordingly, facial action unit approaches comparing differences in facial shapes between two or more static images (e.g.^6,24^), may struggle to capture global changes in expression. This is particularly relevant to shape changes which are not specific to action units per se, but may nevertheless influence the visual appearance of the face, such as its general outline.

It is argued that the temporal structuring of facial shape changes form an important component in the detection of discrete emotional expressions^25^. This includes the speed of onset and offset of individual action units, and their onset and offset in relation to others^25^. In humans, the reliable differentiation of fear and surprise, and their perceived intensity, is limited when facial images are coded statically rather than dynamically^26^ (cited in^25^). This highlights another potential limitation of relying on static scales such as photographs^4,8,13,14^ and illustrations^9–11^ to visually identify the presence or absence of certain expressions in non-human animals. Additionally, the FACS approach does not readily support the reliable capturing or quantification of the intensity within an expression. This is a pertinent issue where FACS approaches are applied to non-human animals, due to their potential morphological diversity (e.g. that caused by selective breeding). Such diversity may alter the relative ‘neutral’ position of features, and thus corresponding apex, making relative intensity of an expression very difficult to gauge at an individual level, even though it may be of clinical importance.

Another approach has applied ‘traditional’ morphometric methods to quantify facial changes, focusing on linear distances between specific facial landmarks across ‘pain’ and ‘no pain’ conditions (e.g.^10^). ‘Cartoon-like’ hand-drawn images of cat facial expressions were then produced as a means of visualising statistically significant outputs. However, the extent to which these demonstrate reliable and mathematically relevant shape changes was not evaluated. Additionally, these methods are inherently sensitive to the accuracy of landmark placement, as well as the scale, orientation, and, (particularly where images are being collected from live specimens), the pose present within an image^27,28^. If such factors are not sufficiently addressed (e.g.^10,11^), then the results are not necessarily robust or reliable.

The use of linear measurements in this context is also intrinsically limited as they do not provide information on the relative positions of the points between which they are taken, and, as a result, identical linear measurements may characterize very different overall shapes. In addition, even non-linear measurements may (and frequently do) correlate strongly with size, which varies substantially within certain species^29–32^. For instance, two very different objects may exhibit identical ratios between two linear dimensions, even though the extreme points delimiting such dimensions show very different reciprocal positions. Their subsequent representation in any interpretation, is then at risk of subjective bias. Furthermore, these traditional measurements do not allow for graphical representation of shape differences built from them. This is because the shape of the original specimen cannot be recovered from the associated data matrices produced when using linear distances^33^. Finally, distance-based measurements do not readily support the exploration of facial lateralisation, even though previous evidence would suggest this may be important in relation to the facial expression of affective states such as pain (see^9,34–36^).

Landmark-based geometric morphometric methods obviate these limitations and offer a much more powerful and discriminatory tool for quantifying shape and its variation^33,37^. Geometric morphometric analysis uses the configuration of points (landmarks) positioned on objects as proxies for shape. The coordinates (in 2D or 3D) of those landmarks therefore reflect their reciprocal locations, with differences in such locations across objects measuring the amount of shape variation^29–32^. Superimposition methods are used to align coordinates for each ‘specimen’ based on weighted scaling factors, thus eliminating all variation that does not relate to shape, such as the position, orientation and scale of the objects^38,39^. Results of multivariate testing are easily visualised relative to landmark configurations of interest. This facilitates meaningful and practical interpretation of results by mapping the magnitude and direction of movement of relevant coordinates across different populations or other variables of interest^40,41^.

The application of geometric morphometrics to the study of facial expressions is novel, feasible, and timely. Associated protocols are readily available in dedicated free software; the implementations of which allow for much flexibility in data capture, processing, and manipulation. By placing facial landmarks relative to underlying musculature, it is possible to capture dynamic variation specific to different muscle movements, regardless of their recognition as facial action units. Simultaneously, the placement of landmarks at various ‘mid-way’ prominent locations on a face image (e.g., the muzzle, ears and eyes) helps capture more general shape changes. Meaningful aspects of variation can then be mapped visually to facilitate a representation of the way in which the face changes as the result of different conditions, or under different experimental settings.

The domestic short hair (DSH) cat provides a particularly useful model for exploring the use of geometric morphometric approaches, for several reasons. Firstly, data availability on the facial anatomy of the cat, in combination with a detailed repertoire of facial expressions (i.e. a Facial Action Coding System for cats (catFACS))^17,42^, make it possible to map biologically relevant facial landmarks with relative ease. Secondly, due to cat’s hair covering, certain facial features (e.g. wrinkling) commonly used in the classification of facial expressions in humans^15^, are much less visible. As such, there is a need to develop methods that do not rely on these types of features being evident. Thirdly, DSH cats have a reasonable degree of variation in facial morphology, without the additional complication of a highly variable and largely protruding muzzle (such as mixed-breed domestic dogs for example). This provides an opportunity to test the robustness of the geometric approach within a relatively diverse population, in species whose facial landmarks can be easily mapped from 2D images.

Therefore, the aim of this study was first to quantify changes in the facial expressions of cats in relation to a painful experience using a geometric approach, and then to validate these changes against a “gold standard” composite measure of post-operative pain (UNESP-Botucatu MCPS tool)^43^.

## Methods

The dataset relating to the cats used for this study was collected previously for the purposes of validating a composite pain scale in domestic cats, not specifically involving the face (see^43^), and its use was approved by the Institutional Animal Research Ethical Committee of the FMVZ-UNESP-Botucatu under the protocol number of 20/2008. The use of this dataset for the assessment of pain within the current project was approved by the delegated authority of the University of Lincoln, School of Life Sciences, Research Ethics Committee UID: CoSREC252. All experiments were performed in accordance with relevant guidelines and regulations.

### Identification of suitable facial landmarks

**Figure 1:**
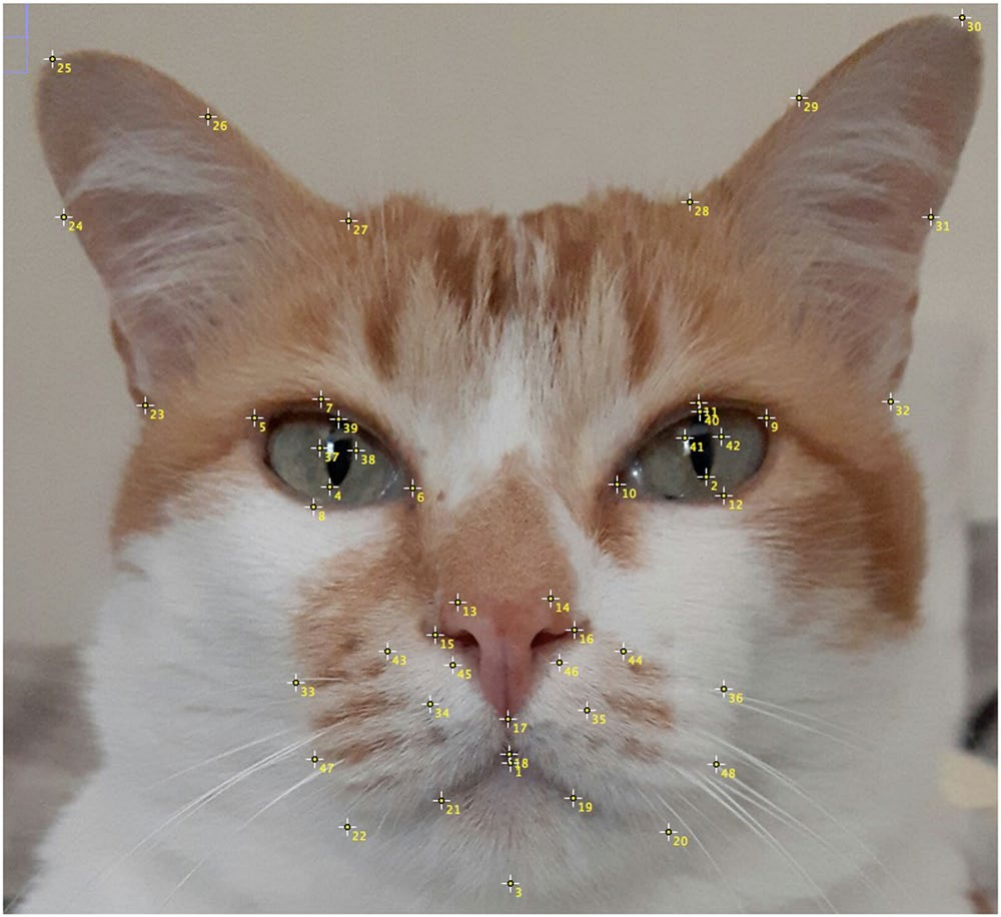
Mirror image of cat’s face, depicting placement of the 48 facial landmarks. Landmarks appear contralateral to their origin, as they would when directly observing the cat’s face. For more information about the landmarks and their placement, see Supplementary Data S1.

Based on both the anatomy of cat facial musculature, and the range of facial expressions generated as a result of facial action units^17,42^, 48 individual facial landmarks were identified to capture as much relevant facial shape change as possible (See Fig. 1 and Supplementary Data S1 for further information on landmark placement and relevance to facial musculature and action units). Landmarks were visually identified and manually annotated by LF. Points consisted of a combination of Type I landmarks (i.e. landmarks that identify the location of muscle insertions which move during contraction), and Type II landmarks (i.e. landmarks that are not specific to insertion points, but capture general facial shape changes, as a result of the occurrence of various muscle contractions).

### Data collection

Material used for the extraction of facial expressions comprised of footage from 29 healthy mixed breed (i.e. DSH) female cats (2.8 ± 0.5 kg; 14.1 ± 5.2 months) undergoing ovariohysterectomy as described in^43^. Cats were recorded at four separate time points over the perioperative period, representing four separate conditions as follows:

T1 - Pre-surgery (between 18–24 hours during the preoperative period), T2 - 1 hour post-surgery (between 30 minutes and 1 hour after the end of surgery, and prior to administration of additional analgesics), T3 - Post-rescue analgesia (approximately four hours after postoperative analgesia), and T4 - 24 hours post-surgery (approximately 24 hours after the end of the surgery).

All cats were anesthetized prior to surgery (between T1 and T2) with propofol^a^ IV (8 mg/kg), fentanyl^b^ (0.002 mg/kg) IV and isoflurane^c^ in 100% oxygen. Postoperative analgesics (Morphine^d^ (0.2 mg/kg) IM, ketoprofen^e^ (2 mg/kg) SC and dipyrone^f^ (25 mg/kg) IV) were administered to all cats approximately 1 hour after the end of the surgery (between T2 and T3), after video recording for T2 was completed. Each cat was given a multidimensional composite pain scale (MCPS) score at each time point^43^. The MCPS is a well validated measure of postoperative pain for cats undergoing ovariohysterectomy which includes a range of behavioural measures associated with pain assessment (e.g. posture, comfort, activity, attitude, response to palpation, demeanour, appetite), but which does not focus specifically on the face^43^.

### Image extraction and annotation

Image extraction was, as far as possible, limited to periods of time when cats were not considered to be distracted by any external stimuli in the environment or being physically handled, since both handling^12^ and distraction may alter the perception and physiological impact of noxious stimuli^44,45^. Since averaged measures derived from multiple contributions of each individual provide a more reliable representation, multiple images from each cat, from each video, were sampled on the basis of those considered suitable for annotation (see below for criteria used). An inter-sample interval of at least 4 seconds was used to minimize the risk of non-independence of dynamic expressions^46^ while ensuring a large number of images could be captured of the same face in the given state.

To maximise data collection, both frontal views, as well as those involving a degree of lateralised pose were included, as long as all relevant landmark features could be annotated. The inclusion of such pose was considered acceptable given that (i) all facial coordinates were subsequently subjected to scaling, translation and rotation^47^, and that (ii) emergent sources of shape variation within the dataset could be tested across known conditions linked to variation in pain intensity (see further on in methodology). This method therefore enabled the detection and isolation of pain-based shape variation even in the presence of substantial pose.

A total of 932 individual images from 29 cats undergoing ovariohysterectomy across the four conditions were extracted. Landmarks were manually annotated and digitized using ImageJ: version 1.49v^48^, to create a geometric representation of the cat’s face in the form of a series of 48 paired x-y landmark coordinates. Usable images ranged from 1 to 23 per video, depending on the availability of suitable frames that could be captured.

To establish the reliability of the landmark annotation process, a second person manually annotated approximately 10% of images from the dataset, using Fig. 1, and the materials included within the Supplementary Data S1 for reference. Images used for reliability analysis were selected pseudo randomly, so that contributions were balanced across individuals and conditions. At the point of annotation, both annotators were blinded to the condition from which each image was drawn.

### Statistical analysis

Landmark configurations were extracted from the 932 images. Scaling, translation and rotation effects were eliminated via Procrustes superimposition using MorphoJ^47^. MorphoJ^47^ was then used to perform a PCA on the Procrustes coordinates in order to produce a pattern of shape variation readily visualized in morphospace^47,49^. In order to determine that the structure derived from the PCA was robust, individual cats and their associated data contributions were pseudo randomly divided (to ensure even distribution of subjects between data sets) into two separate subsets, and the PCA repeated on each subset. Inter-annotator reliability for all 96 x-y coordinates was determined via the Inter Class Correlation Coefficient ICC2 (a measure of absolute agreement between raters)^50^. ICCs were calculated using R version 3.4.2^51^.

In order to visualize the magnitude of displacement of different landmarks relative to one another, (and thus where the most obvious shape changes were occurring within the face for each PC of interest), we used lollipop graphs. These graphs illustrate landmark displacements (using the convention towards the positive direction of a given PC axis), relative to the average configuration of all Procrustes-fitted shapes. The scree plot and proportion of variance explained by each PC was inspected in combination with the lollipop graphs in order to reasonably determine how many PCs to retain in the subsequent analyses. Average PC scores, based on the weighted loading of each coordinate within a given component, were then generated for each of these PCs, for each cat, across each condition.

To assess whether the variations in facial morphology identified in the preceding steps were related to the pain status of individuals, differences in (retained) PC scores across the conditions were then analysed in PAST version 3.10^52^. One-way repeated measures ANOVAs, with post-hoc Tukey tests, and Bonferroni corrected p values were used to identify the source of any significant differences. Principal components were retained where significant differences in their scores across the conditions (in which pain intensity was predicted to vary) were identified. Corresponding lollipop graphs were examined and interpreted relative to the shape variation they represented. The average facial landmark positions for each condition (T1–4) were plotted for visual comparison.

To assess the unimodality of principle component scores, density distributions of the PC values retained in the preceding step were determined overall and then per condition (T1–4), via Hartigan’s dip tests^53^, using the ‘diptest’ package in R. To assess the discrepancy (separation) between density functions associated with PC values across the four conditions, differences in probability density distributions of the retained PC scores were then analysed. A series of pairwise comparisons across the four time points were performed, using the kernel density based global two-sample comparison tests^54^, via the kde.test function in the ks package in R.

To assess whether Botucatu MCPS scores varied across conditions, total pain scores for each cat were calculated based on the sum of their subscales^43^. These were then subjected to one-way repeated measures ANOVAs and post hoc Tukey tests, using Bonferroni corrected p values. Only cats that had extractable images across all four conditions (T1–4) were used in these ANOVAs.

Validation was assessed from the relationship between average (retained) PC scores and Botucatu pain scores for the same cats used in the ANOVAs. These were analysed using general linear models in R version 3.4.2^51^, with PC scores as the response variable and Botucatu scores and condition (and their interaction) as the explanatory variables. The assessment of the impact of the interaction between Botucatu scores and condition upon PC scores was included so that the consistency of the relationship between Botucatu and PC scores across conditions could be determined. Variables were standardised by converting to Z scores prior to analysis. Data were checked for normality prior to analyses and where appropriate, subsequent model diagnostics were performed to assess heteroscedasticity and check for overdispersion. For all relevant statistical tests, an alpha of 0.05 was used.

A general overview of the key aspects of the methodology, their purpose, and associated outcomes is provided as a flowchart in supplementary material (Fig. S1).

## Results

### Principal components and relative shape variation

The first eight principal components explained 87% of the variation within the population, with individual contributions ranging from 35% (PC1) to 2% (PC8), and subsequent components each contributing ≤1% (see Supplementary Data S2 for relevant statistical outputs). Lollipop graphs indicated shape variations with varying degrees of lateral differences across the PCs. All eight components were initially retained and included in subsequent ANOVA’s, and post hoc tests where relevant.

The output of the PCA was robust, with the structure confirmed in the two subsets (see Supplementary Datas S3 and S4). Additionally, lollipop graphs generated for the PCs extracted from these subsets indicated very similar changes in face shape (e.g. magnitude and direction of landmark displacement), compared to those retained in the full dataset (see Supplementary Fig. S2). ICC’s for each of the 96 x-y coordinates were generally very good or excellent, ranging from 0.69 to 0.99, with a median value of 0.9 (see Supplementary Data S5).

### Differences in PC scores across conditions

25 cats had extractable images across all four conditions (T1–4). Across the eight PC components tested, only PC3 (F_3,96_ = 10.9, p < 0.0001), PC6 (F_3,96_ = 3.101, p < 0.03195) and PC7 (F_3,96_ = 3.147, p < 0.0302) scores showed significant differences across the conditions. However, post hoc analyses revealed that for only PC3, were average scores significantly different between all time points where distinct differences in pain intensity were predicted.

**Figure 2:**
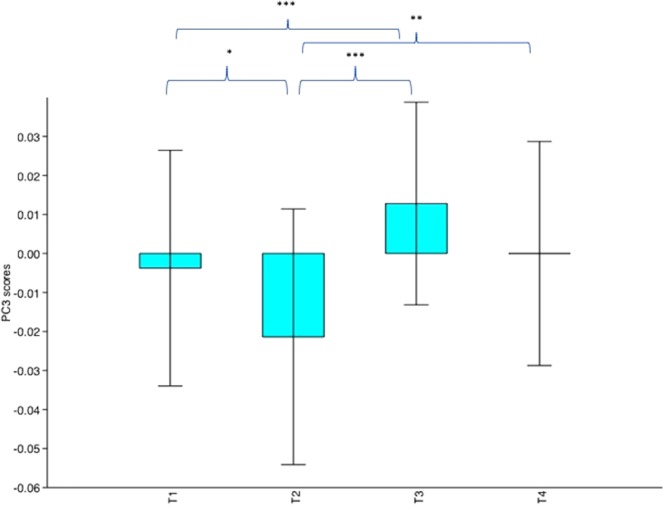
Mean PC3 values with standard deviation for cats undergoing surgery with analgesia (n = 25). T1 = before surgery, T2 = 1 hour post-surgery, before rescue analgesia, T3 = post rescue analgesia and T4 = 24 hours post-surgery. *p < 0.05, **p < 0.01, ***p < 0.001.

**Figure 3:**
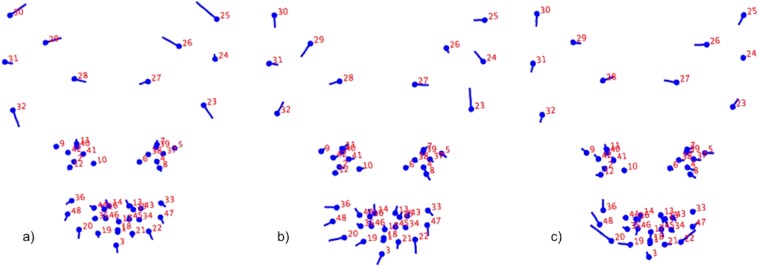
Geometric representation of the face based on average landmarks from 932 images extracted from 29 cats, created using lollipop graphs. Relative facial shape changes associated with higher PC values for PC3 (**a**), PC6 (**b**) and PC7 (**c**) are represented by the lines protruding from each landmark, highlighting the direction and magnitude of movement. Higher PC scores are therefore reflected by a greater distance along the line from the circular nodes, with lower PC scores reflecting less distance from the nodes. For ease of reference between the PC shape change descriptions (excluding i–vii below), and corresponding images, images have been ‘flipped’ horizontally so that the left side of the cat’s face is located on the left side of the image. Images produced using MorphoJ, Version 1.06d^47^.

**Figure 4:**
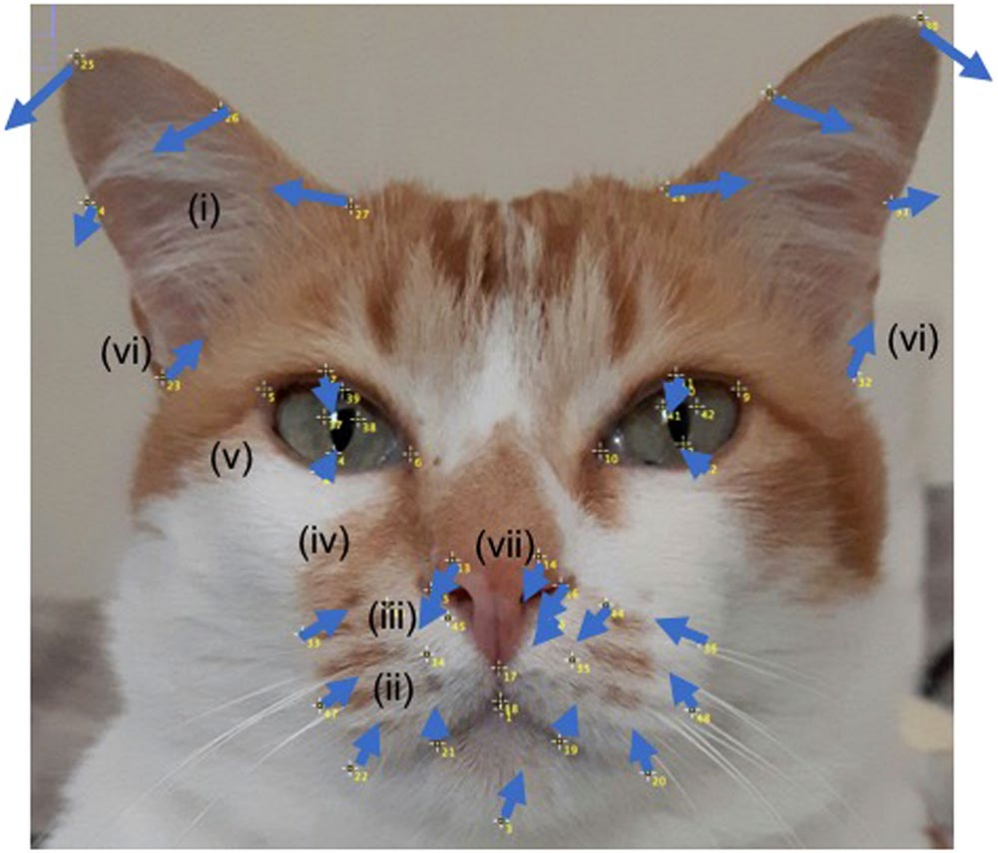
Mirror image of cat’s face, depicting placement of the 48 facial landmarks. Arrows have been added to indicate the direction and magnitude of landmark displacements associated with increased pain intensity, (as described in (i–vii) above). Landmarks appear contralateral to their origin, as they would when directly observing the cat’s face.

Compared with T2 (1 hour post-surgery, prior to rescue analgesia), average PC3 scores were significantly higher at all other time points; at T1 (before surgery), (Q = 4.12, p = 0.02415), at T3 (post rescue analgesia), (Q = 8.002, p = 0.0001507) and at T4 (24 hours post-surgery), (Q = 4.998, p = 0.004032). PC3 scores were also significantly higher at T3 compared with T1 (Q = 3.882, p = 0.03757). Average PC3 scores for T1 and T4 and T3 and T4 did not differ significantly (both p > 0.05), see Figs 2, 3 and 4. Post hoc comparisons for PC 6 revealed a significant difference between only T3 and T4, with PC6 scores significantly higher at T3 (Q = 3.788, p = 0.04443). Only a near-significant difference between T2 and T3 was evident for PC7, with scores being higher at T2 (Q = 3.679, p = 0.05375).

### PC shape changes

Coordinates relating to ear and muzzle shape loaded most prominently on PC3, with an increase in component score indicating displacement of the ventrolateral edge of each pinna (landmarks 23 and 32) ventrally; in the case of the cat’s right ear, also laterally, and in the cat’s left ear, medially. The pinnae apex (landmarks 25 and 30), and the midpoints (landmarks 26 and 29) of the medial edge of each pinnae were displaced in a dorsomedial direction, and the medial ventral edge of each pinna (landmarks 27 and 28) in a ventromedial direction. Landmarks around the mouth and chin area (landmarks 3, 19–22, 33, 36, 47,48) were generally displaced in a ventral or ventrolateral direction, whilst those on the nose area (13–17, 44–46) indicated a dorsal as well as lateralised displacement to the cat’s left. A comparatively smaller displacement was identified around the eyes (landmarks 7–8 and 11–12), where points on the upper and lower medial eye points were displaced either dorsally (7 and 11) or ventrally (8 and 12), see Fig. 3.

**Figure 5:**
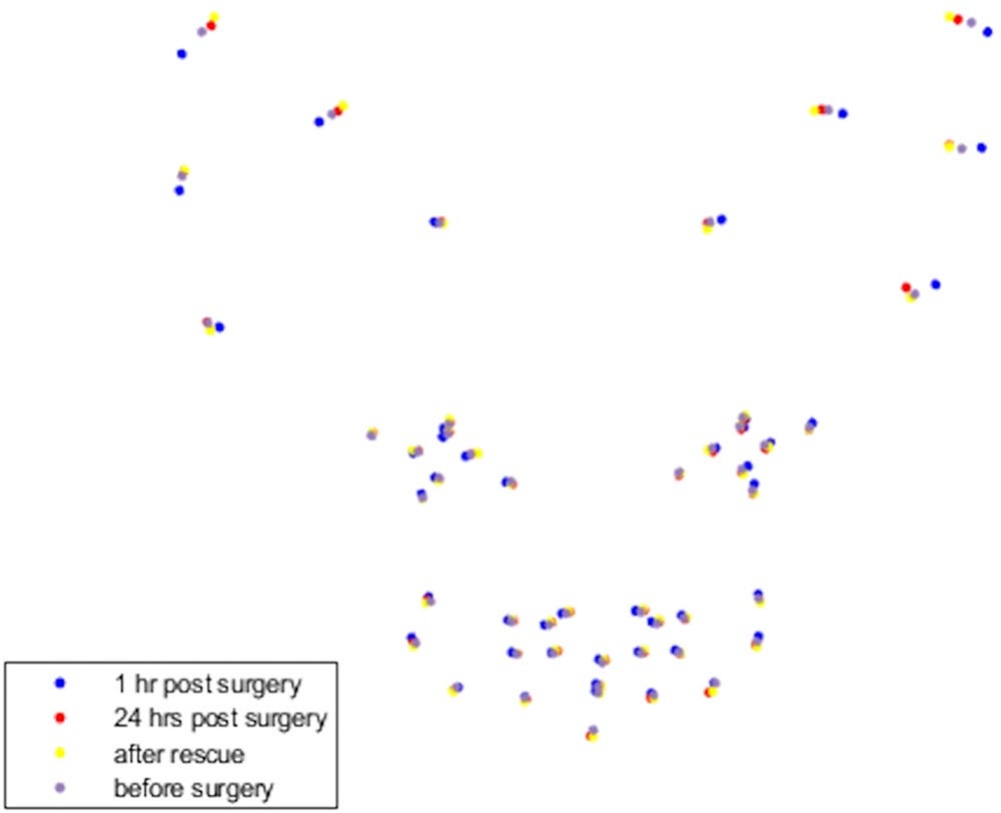
Geometric representation of the face based on average landmarks across the four conditions (T1–T4) for cats undergoing surgery with analgesia (n = 25).  = before surgery (T1),  = 1 hour post-surgery, before rescue analgesia (T2),  = post rescue analgesia (T3),  = 24 hours post-surgery (T4). Average landmarks reflect mirror image and are positioned contralateral to their origin, so appear as would when directly observing the cat’s face.

Collectively, these displacements indicate a lower PC 3 score (associated with greatest pain intensity, i.e. T2) to reflect the following key features. For ease of reference, these key features (i–vii) are described in relation to how they would appear visually when directly facing the cat (therefore contralateral to their origin). A visual representation of these face shape changes is represented in Fig. 5. A dynamic version is also included in the supplementary materials (Video S1).

(i) A more lateral and ventral positioning of the ears.
(ii) A more dorsal and medial positioning of the cheek and mouth area.
(iii) A reduced distance between the cheeks, mouth and nose region.
(iv) A reduced distance between the cheeks, mouth and the eyes, but an increased distance between the nose and eyes.
(v) A slightly narrowed eye aperture.
(vi) Lateralised differences in the ears, associated with a more dorsomedial positioning of the left lateral pinnae edge, and a more dorsolateral positioning of the right lateral pinnae edge.
(vii) A lateralised difference in the nose, with a more left and ventral positioning.

For PCs 6 and 7, co-ordinates relating to the ears, muzzle, and also eyes all loaded prominently, although the landmark displacements generally occurred in different directions. Of the three PCs, PC6 displayed the greatest degree of lateralised displacements. Ear landmarks on the lateral edges of the pinnae (landmarks 23–25, 31–32) were displaced medially, with several of the cat’s right ear points also displaced dorsally (landmarks 23, 24, 27), and those of the cat’s left ear points also ventrally (landmarks 28–31). Lateral differences were present in the eyes, with the cat’s right eye points displaced in a ventrolateral direction (landmarks 4, 5, 8, 37), and the cat’s left eye points displaced in a medial or dorsomedial direction (landmarks 9–11, 40, 41). The mouth and cheek area indicated displacement in a ventral direction, with a lateral displacement on the cat’s left side of the face (landmarks 3, 19, 20, 36, 47, 48). Points of the nose were displaced in a dorsolateral direction which again was stronger on the cat’s left side (landmarks 13, 14, 35, 43, 44, 46).

The most prominently loading landmarks for PC7 were those around the muzzle region, with areas of the cheeks being displaced in a dorsolateral direction, again strongest on the cat’s left side (landmarks 20, 22, 36, 47, 48). The points along the medial edges of both pinnae were displaced in a dorsomedial (landmarks 27 and 28) and ventromedial direction (landmarks 26 and 29), with the points located on the lateral edge of the cat’s left pinnae displaced in a ventrolateral direction (landmarks 30–32). Finally, the eye points were generally displaced laterally (landmarks 4, 5, 7–9, 11, 12, 37–39, 42), with the ventral points of the cat’s right eye also ventrally (landmarks 4 and 8), and the dorsal points of the cat’s left eye (landmarks 9, 11 and 42) dorsally (see Fig. 3).

### Density distributions and discrepancies in density functions

Density distributions for PC3 scores were unimodal, both overall as well as for each condition (T1–4). Of all pairwise comparisons between conditions, the only significant difference in density functions for PC3 scores were between T2 (1 hour post-surgery, before rescue analgesia) and T3 (post-rescue analgesia), (p = 8.147882e-11).

### Differences in BOTUCATU pain scores across conditions

**Figure 6:**
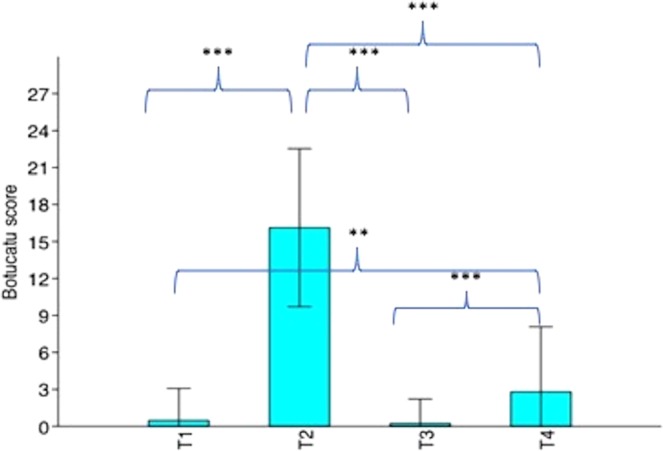
Mean Botucatu pain scores with standard deviation for cats undergoing surgery with analgesia (n = 25). T1 = before surgery, T2 = 1 hour post-surgery, before rescue analgesia, T3 = post-rescue analgesia and T4 = 24 hours post-surgery. *p < 0.05, **p < 0.01, ***p < 0.001.

Botucatu pain scores were significantly different amongst the four conditions (F_3,72_ = 285.1, p < 0.0001). Compared with T2 (1 hour post-surgery, pre-rescue analgesia), scores were significantly higher at all other time points; T1 (pre-surgery), (Q = 34.92, p = 0.0001), T3 (post-rescue analgesia), (Q = 35.46, p = 0.0001), and T4 (24 hours post-surgery), (Q = 29.74, p = 0.0001). Scores at T4 were also significantly higher than at T1 (Q = 5.18, p = 0.0027), and T3 (Q = 5.716, p = 0.0009). There were no significant differences between T1 and T3 scores (Q = 0.5359, p = 0.9814), see Fig. 6. Such results indicate that the smaller data set used in this study compared with Brondani *et al*.^43^ (e.g. n = 25 versus n = 30), did not impact on the consistency of the relationship between Botucatu scores and conditions.

### Relationship between botucatu tool and PC scores

**Figure 7:**
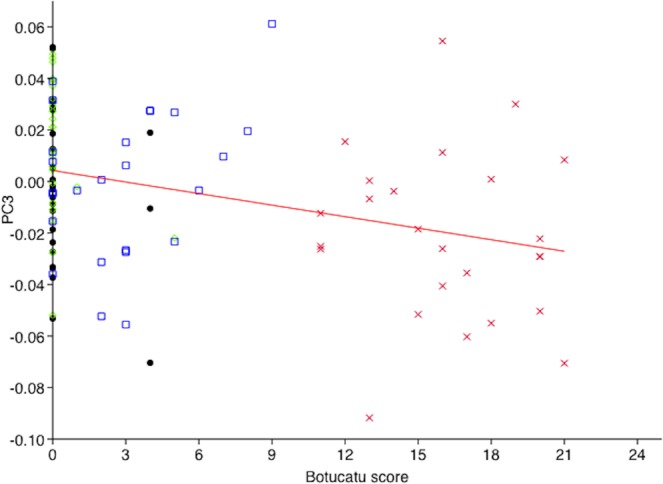
X-y Regression plot of Botucatu and PC3 scores for cats undergoing surgery with analgesia (n = 25). ● = T1 (pre-surgery),  = T2 (1 hour post-surgery, pre-rescue analgesia),  = T3 (post-rescue analgesia),  = T4 (24 hours post-surgery).

A significant relationship between PC and Botucatu scores was identified for PC3 (F_1,98_ = 11.94, p = 0.00082). PC3 scores decreased significantly with an increase in Botucatu score (Fig. 7). There was no significant interaction between Botucatu scores and condition (p < 0.05), indicating a consistent relationship between PC and Botucatu scores across variations in pain intensity (i.e. time points T1–T4).

## Discussion

Our first objective was to geometrically quantify facial expressions in relation to a painful procedure, and this was achieved through the identification of a single principal component (PC3) relating to ear, muzzle, cheek, and to a lesser extent, eye shape variation. This component was found to differ significantly between all conditions where substantial differences in pain intensity were expected to be present. In all cases, PC3 scores were significantly lower in the condition associated with the greatest intensity of pain (i.e. T2). Convergent validity between the facial changes identified and an independent ‘gold standard’ composite measure of post-operative pain (UNESP-Botucatu MCPS tool)^43^ was established through a significant negative relationship between PC3 and Botucatu scores. This relationship was consistent across conditions, as indicated by the lack of a significant interaction between Botucatu scores and condition. Higher Botucatu scores, indicating greater pain, were associated with lower PC3 scores.

Face shape changes across the ears, muzzle and nose in particular were clearly highlighted in the geometric diagram of PC3 landmark displacements (Fig. 3), and these were also reflected at an average population level via subtle shape variations across the conditions (Fig. 4). At this level, the more discernible differences in landmarks were located around the ears (e.g. (i), see also Fig. 5) suggesting that following administration of analgesia (i.e. T3) when pain should be absent, the ears were held in a slightly more upright position, and were held at their lowest position when pain would be expected to be most intense (T2). This relative change in position is consistent with the ‘Ears downward’ action descriptor documented within catFACS, which describes the ears being ‘pulled ventrally, sliding laterally in the head’ and the ‘bases of the pinna moving away from each other’^17^.

Shape differences for the nose, mouth, cheek, and especially eye areas (e.g. (ii–v), see also Fig. 5) were less distinct at the population level, although several differences across conditions were still evident (Fig. 4). The lateral edges of the cheeks/whisker pad area were most dorsally displaced at T2, and ventrally displaced at T3 (with T1 and T4 points overlapping in between). In the case of the eyes, T2 landmarks were generally positioned more closely to one another, creating a smaller eye aperture (with the other time points mostly overlapping). Such displacements may reflect the output of the action unit ‘Upper lip raiser’ described in catFACS^17^, where the bottom and outer edges of the upper lip move dorsally, and in strong movements also obliquely towards the medial eye corner, a movement which in the process changes the relative positioning of the cheeks and area of the whisker pad, also moving them dorsally and obliquely in the same direction as the upper lips.

Within catFACS, the ‘Upper lip raiser’ action unit is always coded together with ‘Nose wrinkler’ in this species due to the concomitant effect of specific muscle contractions on these two facial regions^17^. In strong movements, the dorsal raising of the lip and infraorbital region (located ventral to the eye and lateral to the nose) is described as creating a deep wrinkle along the nose, with the nostril wing raised and widened as the lips move dorsally.

During the process of ‘Upper lip raiser’ and ‘Nose wrinkler’, the area below the infraorbital region is also described as moving dorsally and towards the medial eye corners, causing a narrowing of the eye aperture in strong movements^17^. Such combination of movements appears similar to those identified in PC 3 shape displacements (Fig. 3), (see also (ii-v) and Fig. 5), and to a more subtle extent at the average population level (Fig.4). Collectively, our results therefore suggest that the facial movements of ‘Ears downwards’, ‘Upper lip raiser’ and ‘Nose wrinkler’ identified in the catFACS^17^, may be linked to pain expression in domestic cats.

Lateralised differences in landmark displacements relative to pain intensity were also identified (see (vi–vii) and Figs 3, 4 and 5). Landmarks located around the nose area indicated a (left) lateral and slightly dorsal appearance at T2 (greatest pain intensity), compared to the other time points, which mostly overlapped. Lateral differences were also evident in the ears, with the left-side ear (i.e. the cat’s right) displaying a comparatively more intense lateral and ventral positioning at T2 compared to T3 (Fig. 5).

Various forms of brain and behavioural lateralisation (based on their emotional valence, in response to a range of stimuli), have been documented in a wide range of species (e.g. see following reviews^55,56)^. Pain has previously been associated with increased activity in the right amygdala in rats^35^, and right hemisphere in humans^36^, with a contralateral effect^34^. However, there is currently only limited evidence of lateralisation in relation to the behavioural expression of pain, with no mention of relative directionality. For example, Gleerup *et al*.^9^ report “asymmetries” in the ear carriage of horses, but did not specify if these are lateralised effects. The results of this study therefore provide evidence of lateralisation in facial expressions in relation to pain, although not necessarily in accordance with findings in humans (e.g.^34,36^), unless the relevant facial movements in cats are controlled in an ipsilateral, rather than contralateral way (see^57^).

Lateralisation in shape change was also prominent in PC6, indicating displacement of the mouth and cheek regions to the cat’s left, and displacement of the eyes to the cat’s right, occurring dorsally for the cat’s left eye, and ventrally for the cat’s right eye. Both ear apertures appeared reduced, which may reflect the output of the action unit ‘Ears constrictor’ described in catFACS, in which both edges of the pinnae move towards each other, decreasing the ear aperture^17^. In contrast to the left ear, the apex of the cat’s right ear was displaced medially, and the lateral edges in a dorsomedial direction. This may reflect the output of the action unit ‘Ear rotator’, where the caudal surface of the ear rotates medially, and the lateral edges of the pinna appear relatively more dorsally positioned^17^. Higher PC scores, indicating greater relative displacement, were evident post-rescue analgesia (T3) compared with 24 hours post-surgery (T4). However, no differences in PC6 scores were identified between T2, where pain was expected to be most intense, and any of the other time points (e.g. T1, T3 and T4, where pain was expected to be absent or abated).

Therefore, whilst these lateralised differences may not be reliable facial indicators of pain, it is possible they are associated with other forms of negative affect (for example, a left head and gaze bias has previously been associated with anxiety in cats^46^, and a right ear rotation bias with frustration)^58^. It is also possible that the lateralised differences identified in PC6 simply represent a greater amount of ‘noise’ or ‘pose’ present within images captured at T3 versus T4. This may be reflective of the cat’s face appearing less frequently in a frontal position in relation to the camera during T3, meaning more general pose was present in the images captured. Further field observations should be able to elucidate on these potential sources of lateralisation.

These various subtle occurrences of facial lateralisation are potentially easier to capture and quantify using a geometric rather than ‘Grimace scale’ method, which is the more commonly used approach at present^4,6–11,14^. Our approach therefore lays the foundation for the development of a tool sensitive to lateralised responses, which appears to be an area that has so far received insufficient attention.

Several of the pain-linked face shape changes identified within the current study are also consistent with those previously identified in cats^10,11^ and the wider animal literature^4,6,7,9,13,59^, adding to the external validity of our findings. However, until now, the ability to make general comparisons across studies or species has been limited by a lack of standardisation regarding the way in which facial expressions have been measured and described. In addition, we have identified various displacements around the mouth and nose, as well as potential lateralised differences associated with the nose and ears, which have not been previously reported.

At the population level, differences in average face shapes relative to pain were visually subtle (Fig.4). A repeated measures analysis approach indicated significant differences in PC3 scores between T2 (1 hour post-surgery, pre-rescue analgesia, representing greatest pain intensity) and all other time points (e.g. T1, 3 and 4, where pain is either absent or abated). However, at a population level, analysis of discrepancies in density functions indicated significant differences only between T2 (greatest pain intensity) and T3 (post-rescue analgesia), suggesting that the differences in shape changes between these two time points (see transition from blue to yellow, Fig.4), are those particularly consistent across individuals. This transition might therefore be considered to be the prototypical changes that define the pain face in cats.

The subtlety of this general difference is potentially reflective of the inherent morphological variability within the faces of cats in our DSH or ‘mixed breed’ population, given the large diversity in skull and associated facial morphology that exists at a species level^60,61^. As such, the effect within an individual may be much more pronounced, since skull morphology will be consistent intra-individually. At a population level, these results therefore indicate more the relative direction of change, rather than its extent, with morphological differences in essence shifting the ‘baseline’ and corresponding apex of facial movements for each individual. Whilst such types of variability have yet to be investigated relative to deviations in facial morphology, base line variation in facial expressions have been linked to both strain type and sex in mice^62^, with sex differences also evident in relation to the expression of pain in humans^63^. This highlights the importance of discerning differences seen at the population level, with those seen within individuals. Thus, with good baseline data of the individual before a painful event occurs, the detection of pain at a later date, may be more obvious and straightforward. This may indicate that owners (especially with training on what to look for) may be the best monitors of pain in their cats. The current challenges associated with individual variation in ‘baselines’ therefore need to be considered, but at a clinical level are not insurmountable if animal owners/keepers are able to establish individual baselines for comparison.

It has been suggested that a functional benefit of change in facial expression in response to environmental challenge, is to modify sensory input in a way that supports self-preservation^64^ (cited in^4^). For example in mice, a reduction in eye aperture, and changes in the shape and orientation of the ears and whiskers, may moderate pain perception by limiting external sensory input^4^. Such modifications may also help to avoid potential damage to more vulnerable areas of the face, by reducing their level of exposure (i.e. by reducing eye aperture, or pressing features such as whiskers and ears back against the face). These hypotheses offer a phylogenetically coherent, functional rationale for the homologies seen in facial expressions seen here, and across other species.

The identification of a consistent expression in adult domestic cats is particularly notable given that they, like ferrets^14^, have ancestors which are not socially obligate^65–67^, thus the theoretical benefits of communicating a state like pain, may be more limited (i.e. its expression will not increase the chance of soliciting care from conspecifics). Indeed, the perceived lack of expression of pain in such species has been seen as a major challenge to its proper management^68^.

It is possible that, in domestic cats, the mechanisms underlying the facial responses to pain represent a vestigial communicative remnant of their more social evolutionary past (see^69^). Although the cat’s most recent ancestors are substantially more solitary^65,67,70^, it is unlikely that there would be strong selection against the facial expression of pain (because this is unlikely to be detectable by predators or conspecifics at any distance), but neither would it be highly conserved in the adult. Indeed, during their neonatal period, individuals are likely to benefit from the communication of pain in the form of the additional care it may solicit. As the animal matures, it is also possible that the value of these expressions typically fall, but their prototypical mechanistic basis remains, leading to the more subtle expressions identified within this study (Fig. 4).

Even if the changes are not easily detectable visually, particularly at a population level, we demonstrate that this approach reveals significant differences in facial coordinates in relation to pain, which can enable the development of automatic systems for pain detection in non-human animals. The way that facial shape information is generated using this geometric morphometric approach (i.e. a series of landmark configurations) lends itself well to the application of machine learning algorithms in order to automate the process of pain detection; as has been trialled in humans^2,71,72^, and most recently in sheep^5^. The technique may also offer a novel opportunity for the detection of other emotion states in a wide range of non-human animal species, which unlike the approach used by Lu *et al*.^5^ does not rely on a priori assumptions regarding the way the shape of the face changes relative to emotional state or context.

The future practical, clinical application of this approach for the automatic detection of pain-linked facial expressions, as well as other states of interest, is potentially very feasible. Full automation of this process i.e. from image input, to a pain prediction output, is likely to require the combination of serval key automated processes. These include face shape localisation and landmark detection (^73,74^), as well as image classification (e.g.^75^). Whilst these techniques have been developed, and subsequently refined, for use with human faces, associated algorithms are potentially easily adapted and applied to non-human animal faces (for examples see^5,6,76^).

## Supplementary information

Figures S1 and S2

Video S1

Dataset S1-S5

Dataset S6

Dataset S7

Dataset S8

Dataset S9

## Data Availability

All data generated and analysed during this study are included in this published article (and its Supplementary Files). Data used in PCA analysis: The full data set (n = 932) is available as Supplementary Data S6. Data for sub population 1 (n = 483) and sub population 2 (n = 449) are available as Supplementary Datas S7 and S8 respectively. Data used for ANOVAs and density distribution analysis are available as Supplementary Data S9.
