# Supplementary material for: Geometric morphometrics for the study of facial expressions in non-human animals, using the domestic cat as an exemplar
Source: Sci Rep. 2019 Jul 8;9:9883. doi: 10.1038/s41598-019-46330-5 (PMC6614427; doi:10.1038/s41598-019-46330-5)
Supplement: Supplementary file 1 — Figures S1 and S2 [file 41598_2019_46330_MOESM1_ESM.pdf]

Geometric morphometrics for the study of facial expressions in non-human animals, using the domestic cat as an exemplar

Lauren R Finka, Stelio P Luna, Juliana T Brondani, Yorgos Tzimiropoulos, John McDonagh, Mark J Farnworth, Marcello Ruta, Daniel S Mills

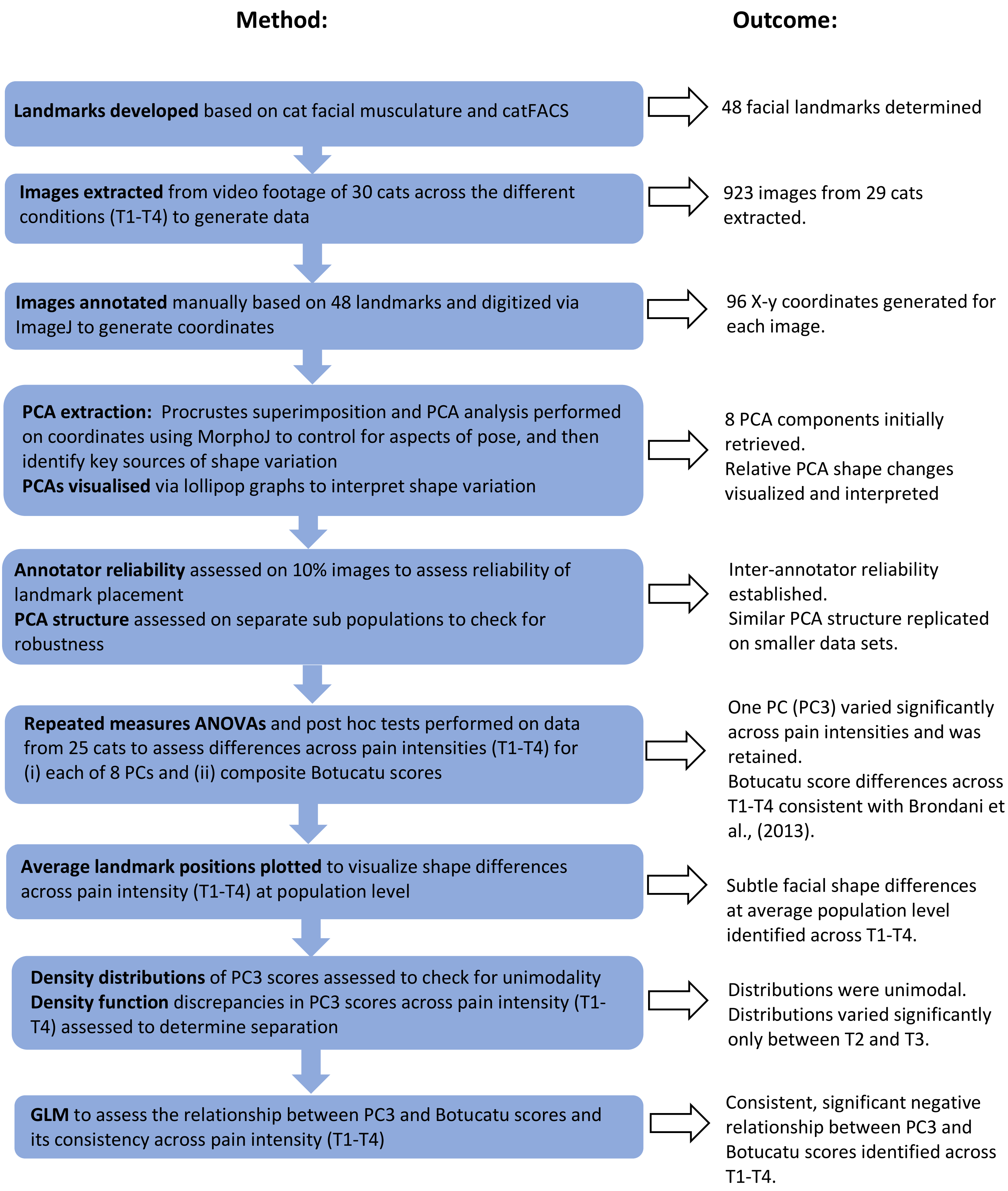

Figure S1. General overview of the methodology. Key aspects, their purpose, and outcomes are indicated.

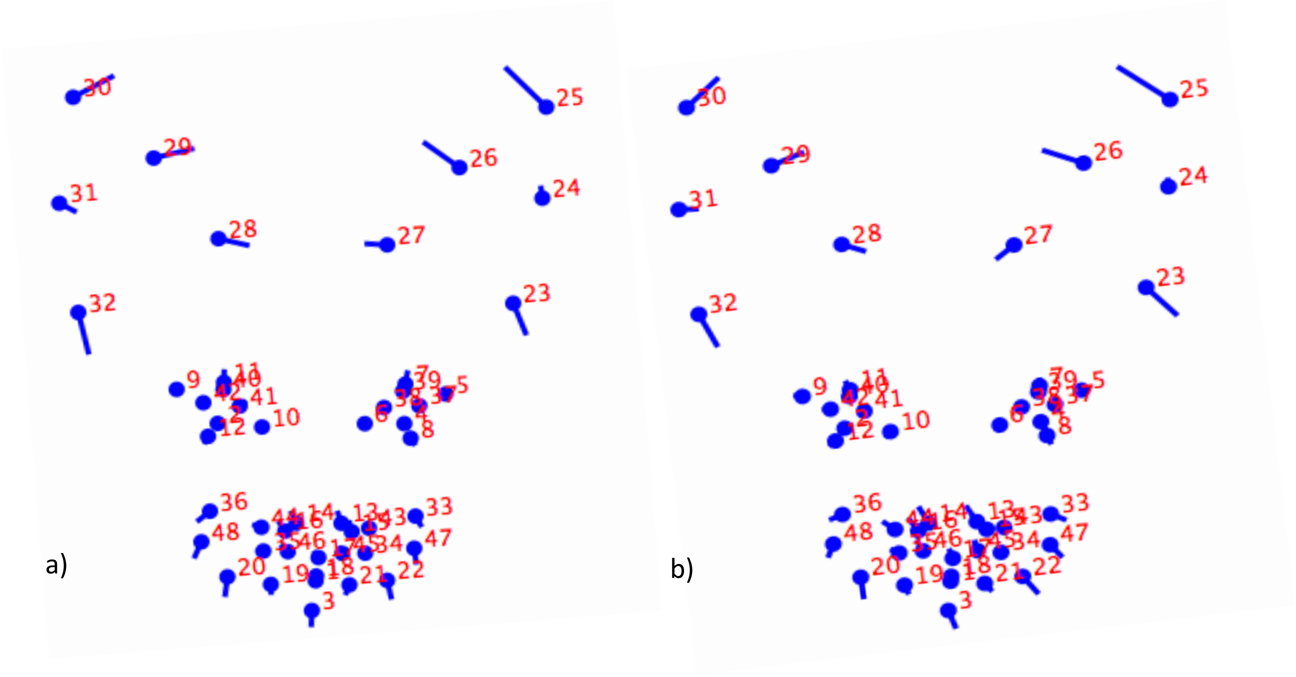

Figure S2. Lollipop graphs of PC3 shapes generated from data included in subset 1 (a) and 2 (b). Images have been ‘flipped’ horizontally so that the left side of the cat is located on the left side of the image. Images produced using MorphoJ, Version 1.06d.
